# Prevalence and risk factors of work-related musculoskeletal disorders among Radiographers: a proposed systematic review and meta-analysis protocol

**DOI:** 10.1186/s13643-025-03017-5

**Published:** 2025-12-12

**Authors:** Ullas U. Nayak, Bincy M. George, Sidhiprada Mohapatra, Vennila J, G. Arun Maiya, Mohandas Rao KG

**Affiliations:** 1 Division of Anatomy, Department of Basic Medical Sciences, Manipal Academy of Higher Education, Manipal, Karnataka India; 2 Division of Anatomy, Department of Basic Medical Sciences, Manipal Academy of Higher Education (MAHE), Manipal, Karnataka India; 3 Department of Physiotherapy, Centre for Comprehensive Rehabilitation, Manipal College of Health Professions, Manipal Academy of Higher Education, Manipal, Karnataka India; 4 Statistics Department, Manipal College of Health Professions, Manipal Academy of Higher Education, Manipal, Karnataka India; 5 Department of Physiotherapy, Centre for Podiatry & Diabetic Foot Care and Research, Manipal College of Health Professions (MCHP), Manipal Academy of Higher Education (MAHE), Manipal, Karnataka India

**Keywords:** Medical imaging professionals, WRMSDs, Workplace injuries, Musculoskeletal disorders, Occupation health

## Abstract

**Background:**

Work-related musculoskeletal disorders (WRMSDs) are significant health concerns among Radiographers, arising from ergonomic challenges, repetitive tasks, and the physical demands of their profession. These conditions contribute to pain, disability, and reduced productivity, emphasizing the need for targeted prevention and management strategies. Despite the growing recognition of WRMSDs, no systematic reviews or meta-analyses have comprehensively synthesized the prevalence and associated risk factors among Radiographers.

**Method:**

This systematic review and meta-analysis will investigate the prevalence of WRMSDs and identify key risk factors in Radiographers. Six electronic databases will be searched from inception to June 2025, using predefined inclusion criteria. The methodological quality of the included studies will be assessed using the Joanna Briggs Institute (JBI) critical appraisal checklists. Pooled prevalence estimates and risk factor analyses will be performed using meta-analytic techniques, with heterogeneity assessed using Cochran’s Q test and I^2^ statistics.

**Discussion:**

The findings of this review will give critical insights into the burden and underlying causes of WRMSDs within this occupational group, emphasizing the need for supporting evidence-based interventions and workplace modifications to enhance their health and productivity. Additionally, this review is expected to inform policy development in the healthcare sector, guiding the implementation of ergonomic equipment design, workplace health promotion strategies, and targeted training programs. By addressing these challenges, the review will support a comprehensive approach to enhance occupational health and fostering a safe, more efficient work environment for Radiographers.

**Systematic review registration:**

PROSPERO, CRD42024565835.

**Supplementary Information:**

The online version contains supplementary material available at 10.1186/s13643-025-03017-5.

## Background

Work-related musculoskeletal disorders (WRMSDs) are injuries or illnesses affecting the musculoskeletal system caused or exacerbated by occupational activities [1]. They are a major contributor to the global burden of disability, with significant economic and social consequences. Musculoskeletal disorders (MSDs) accounted for approximately 150.08 million disability-adjusted life years (DALYs) globally in 2019, with low back pain contributing to 36.8% of cases. The burden of MSDs is rising, particularly in developed countries, driven by occupational risks, lifestyle factors, high body mass index(BMI), and smoking. Women experience a disproportionate burden, with 1.45 times more DALYs than men [2].

WRMSDs are prevalent across various occupational groups, including allied health professionals (AHPs), with reported 12-month prevalence rate ranging from 28 to 96%. Regions such as Africa and Europe reported higher prevalence rates compared to Asia and Americas, reflecting regional disparities in the occupational hazards faced by healthcare professionals. WRMSDs are complex multifaceted conditions influenced by a combination of physical, occupational, environmental, and individual factors, requiring comprehensive strategies for their prevention and management [3–5].

Among these AHPs affected are Radiographers, also known as X-ray technicians or medical imaging technologists, who are healthcare professionals specialized in diagnostic imaging procedures. Their work involves handling heavy imaging equipment, adopting awkward postures during patient positioning, and maintaining prolong visual attention during image acquisition and reporting [6]. Additionally, Radiographers often face the challenge of balancing the need to deliver accurate and timely reports with the realities of crowded scanning rooms and the significant time required for each scan, creating a demanding and stressful work environment that heightens their occupational strain [7, 8].

Recent studies from various geographical regions reported an elevated prevalence of WRMSDs among Radiographers. A study from Western Switzerland found a 12-month WRMSD prevalence of 94.7%, with the neck and low back being the most affected areas. Awkward postures, work stress and being female Radiographers were key risk factors [9]. In the United States, 81% of Radiographers experienced pain or discomfort while handling patients, with significant predictors including poor perceived health, fluoroscopy work and psychological stress [10]. In India, a predictive model developed from a cross-sectional survey identified a 12-month prevalence of 75.2%, with sustained postures, prolonged standing, underweight BMI, and fixed work schedules significantly associated with WRMSD symptoms [11]. A qualitative study from Ghana also reported high rates of low back and neck pain, which affected clinical task performance and contributed to absenteeism and presenteeism. Factors such as lack of assistance, inadequate workforce planning and poor workplace ergonomics were also associated with the development of WRMSDs [12].

These studies collectively highlight the widespread and multifactorial origins of WRMSDs. These disorders not only lead to chronic pain, reduced functional capacity, and absenteeism but also, in some cases early career attrition, all of which will compromise the quality and efficiency of diagnostic imaging services [8]. Although the high prevalence and occupational risk are well documented in individual studies, there is lack of comprehensive synthesis of global data on the prevalence and risk factors of WRMSDs among Radiographers.

Understanding the epidemiology of WRMSDs in Radiographers is crucial for identifying modifiable risk factors, designing effective prevention strategies, and improving workplace ergonomics to mitigate their associated economic and health burdens [7]. Therefore, a systematic review and meta-analysis is essential to address these gaps by synthesizing evidence on the prevalence and factors affecting WRMSDs among this professional group. The findings will provide a robust evidence base to support workplace interventions, ergonomic modifications, and targeted training programs, thereby enhancing the health, safety and productivity of the radiography workforce.

### Review question

What is the prevalence of WRMSDs among Radiographers, and what are the associated risk factors?

The objective of this study is to systematically review and synthesize evidence on the prevalence of work-related musculoskeletal disorders (WRMSDs) among Radiographers and to identify factors contributing to their development.

## Methods

### Protocol registration and reporting

This systematic review protocol is reported in accordance with the Preferred Reporting Items for Systematic Review and Meta-Analysis Protocols (PRISMA-P) statement [13, 14]. The protocol of this systematic review has been registered in the Prospective Register of Systematic Reviews (PROSPERO) database (CRD42024565835).

### Eligibility: inclusion criteria

1) Population: Registered or certified Radiographers who perform diagnostic tests like X-ray, CT and MRI. 2) Exposure: Participants exposed to working conditions of diagnostic radiography environment. 3) Outcomes: Studies must clearly define the WRMSDs of interest such as low back pain, carpal tunnel syndrome or report on any WRMSD affecting relevant body part like neck, shoulder. 4) Risk Factors: Studies investigating risk factors associated with WRMSD like individual, occupational, psychosocial or environmental factors. 5) Study design: All empirical studies will be included, encompassing quantitative observational designs (e.g., longitudinal cohorts, cross-sectional studies, case studies, case series, and case–control studies) as well as qualitative or mixed-methods studies that explore perceived risk factors or experiences related to WRMSDs among Radiographers. 6) Language and Publication: Only peer-reviewed articles published in English, with no restrictions on geographic area, will be included. The review will cover studies published up to June 2025.

### Eligibility: exclusion criteria

Studies not focusing on Radiographers as the primary study population, studies not investigating WRMSDs or their associated risk factors and those published in languages other than English will be excluded. Non-empirical publications such as letter to editors, commentaries, conference proceedings, book chapters, unpublished thesis work, and preprints will also be excluded.

### Data sources and search strategy

A thorough literature search will be performed across six databases: MEDLINE, Scopus, The Cochrane Library, Embase, Web of Science and the Cumulative Index to Nursing and Allied Health Literature (CINAHL) from inception to June 2025. The search strategy will follow Population, Exposure, Outcome, and Study design (PEOS) framework. The search strategy will involve a combination of MeSH terms, Boolean operators, and truncation techniques to ensure comprehensive retrieval of relevant studies. The strategy will initially be developed for MEDLINE and then adapted for other databases, to align with their specific indexing systems and search functionalities. The final search strategy will be reviewed and refined by all authors and in consultation with medical librarian to ensure its accuracy and relevance to the study objectives.

### Study selection

Following the literature search, the identified articles will be imported to RAYYAN AI literature review software and de-duplication will be done [15]. Title and abstract screening will be done independently performed by three reviewers (UN, SM and MR) using predefined inclusion and exclusion criteria. The screening process will be blinded to minimize bias. Conflicts will be resolved by a consensus meeting.

### Quality appraisal

The quality assessment will be performed using Joanna Briggs Institute (JBI) critical appraisal checklists [16]. For prevalence studies, the “JBI checklist for prevalence study” tool will be used; for field test studies, the “JBI checklist for analytical cross-sectional studies” tool will be used, and for mixed method studies, the “JBI checklist for qualitative research” tool will be used [17–19]. To quantify the study quality a quantitative scoring system will be implemented, assigning 1 point for each ‘Yes’ response and 0 for ‘No’ or ‘Unclear’. Scores will be converted to percentages for comparison across studies. Studies will be classified as high quality (> 70%), moderate quality (50–69%), or low quality (< 50%) [20]. This facilitates consistent scoring across various JBI quality assessment tools used, enabling reliable comparisons between studies while preserving the original checklist criteria.

### Data extraction

The data extraction sheet will be developed by (UN), (SM) and (MR). The data sheet will be pilot tested on 25% of the included data and will then be refined. The data extraction sheet will contain- Study details: first author, year of publication, country, journal, title, study design, sample size, and prevalence rate; Participant characteristics: age, gender, work experience and employment setting; Exposure and outcomes: type and anatomical site of WRMSDs (e.g., neck, shoulder, lower back, wrist/hand), frequency and severity of reported injuries, duration or recurrence of symptoms annual; Factors affecting: individual factors (e.g., BMI, physical fitness, comorbidities, previous injury), occupational factors (e.g., workload, posture, manual handling, imaging modality used), psychosocial factors, and environmental factors; Outcome measures: tools or scales used to assess WRMSDs or related risk factors; Findings and methodological notes: key findings, strengths and limitations, and author recommendations. The primary outcome of interest will be 12-month prevalence of WRMSDs among Radiographers and the secondary outcomes will focus on identifying risk factors associated with it (individual, psychosocial, workplace-related). The data will be extracted by single author (UN) and will be reviewed by (MR) (BG) and (AGM) and any doubts and clarifications will be sought by discussion.

### Statistical analysis

All the statistical analysis will be performed using statistical software R 4.2.3 [21]. The prevalence of WRMSDs among Radiographers will be pooled using both fixed and random effect model. The random effects model will be chosen as the primary analytical approach to account for anticipated variability both within and between studies, providing more generalized estimates. For studies that do not directly report prevalence, available data (e.g., number of cases and total sample size) will be used to calculate proportions. When studies present other effect measures (e.g., odds ratios, incidence rates), conversions to prevalence or comparable metrics will be attempted using appropriate statistical transformations following recommended meta-analytic procedures.

Heterogeneity among studies will be assessed using Cochran’s Q test and the I^2^ statistics [22, 23]. A *p*-value < 0.05 in Cochran’s Q test will indicate significant heterogeneity, while I^2^ values ≥ 50% will be interpreted as substantial heterogeneity, and values < 50% will reflect low heterogeneity. To explore sources of heterogeneity, both subgroup analyses and sensitivity analyses will be conducted. Subgroup analyses will be planned a priori based on clinically and methodologically relevant factors, such as: participant characteristics (e.g., gender distribution, years of experience, employment setting), study design (cross-sectional, cohort, case–control), and type or site of WRMSD (e.g., lower back, shoulder, wrist/hand). Sensitivity analyses will be performed to examine the robustness of pooled estimates by excluding studies with high risk of bias, small sample sizes, or outlier values.

Publication bias will be assessed both visually and statistically. Funnel plots will be generated to examine symmetry, and Egger’s test will be used to evaluate bias, with a *p*-value < 0.05 indicating significant asymmetry [24]. The pooled prevalence of WRMSDs will be reported with corresponding 95% confidence intervals (CIs), and where possible, separate estimates for specific body regions affected will be reported.

For studies that cannot be quantified, a narrative synthesis will be conducted following the SWiM (Synthesis Without Meta-analysis) guidelines, emphasizing study characteristics, patterns, and consistency of findings related to WRMSD prevalence and risk factors [25].

## Discussion

This protocol is a critical step toward consolidating evidence on the prevalence and risk factors of WRMSDs among Radiographers. It aims to provide comprehensive synthesis of existing research by summarizing the global prevalence of WRMSDs and identifying the factors contributing to their development. The use of six databases and comprehensive search strategies reflects best practices in systematic reviews, enhancing the likelihood of capturing relevant studies and ensuring a robust synthesis of the existing evidence.

Unlike reviews limited to a single study design, this review will include diverse observational study designs that report factors contributing to WRMSDs among Radiographers. This broad inclusion ensures a comprehensive representation of the current literature and addresses critical gaps in understanding the unique challenges faced by this professional group. The quality of included studies will be assessed using the appropriate JBI critical appraisal checklists for each study design. Facilitate comparison across studies, the responses from the JBI checklists will be quantified using a simple scoring system, assigning one point for each “Yes” response and zero for “No” or “Unclear”. Total scores will be converted to percentages and categorized as high, medium or low quality. This approach maintains the integrity of the original JBI tool while enabling a consistent, transparent evaluation of methodological quality across diverse study designs [20].

While this protocol provides a comprehensive framework, certain limitations and challenges need to be acknowledged. The study is restricted to English-language publications, which may result in the exclusion of valuable information from non- English-speaking regions with potentially high WRMSD prevalence. This limitation could introduce language bias, affecting the global applicability of the findings.

Additionally, subgroup analyses are planned to explore specific factors such as geographic region, years of work experience, type of imaging technology used and work environment. However, the availability and quality of data in primary studies may pose significant challenges. Many studies may lack detailed or standardized reporting on these variables potentially limiting the depth and robustness of subgroup analyses. This variability could contribute to heterogeneity, making it complicated to synthesize results and draw meaningful conclusion from them. In cases where original data are not accessible, verifying data accuracy and consistency becomes difficult, potentially affecting the reliability of pooled estimates. Furthermore, the inability to access raw data may limit opportunities for adjustment or deeper analysis to address confounding factors comprehensively.

Despite these limitations, this protocol adopts rigorous methodological approaches, including structured quality appraisal processes, sensitivity analyses, and subgroup analyses, to mitigate these challenges where possible. These methods enhance the reliability of the findings and provide a transparent foundation for evidence synthesis.

The findings from this review are anticipated to offer critical insights into the burden and determinants of WRMSDs among Radiographers. They are expected to inform the development of targeted workplace interventions, ergonomic improvements, and specialized training programs aimed at reducing the prevalence and impact of WRMSDs. Moreover, the review will support healthcare administrators and policymakers in designing sustainable occupational health strategies that prioritize the well-being of Radiographers. By addressing these challenges and presenting actionable recommendations, this review will contribute to a safer and more productive working environment for Radiographers while promoting overall healthcare efficiency.

## Systematic review registration

The protocol of this systematic review and meta-analysis has been registered in the international prospective register of systematic reviews (PROSPERO) of the National Institute of Health Research available at https://www.crd.york.ac.uk/prospero/ with PROSPERO registration number: CRD42024565835. This review will include all empirical studies including observational designs (longitudinal cohorts, cross-sectional studies, case studies, case series, and case–control studies) and published from inception to June 2025. Any amendments to the review protocol and progress will be documented in PROSPERO. Upon completion, the final manuscript will be submitted to a peer-reviewed journal for dissemination.

## Supplementary Information

Additional file 1: PRISMA-P 2015 Checklist.

Additional file 2: Example search used for identification of articles on PubMed database.

## Data Availability

Not applicable.

## Abbreviations

WRMSDs: Work-related musculoskeletal disorders
MSDs: Musculoskeletal disorders
JBI: Joanna Briggs Institute
DALYs: Disability-adjusted life years
BMI: Body mass index
AHPs: Allied health professionals
PRISMA: Preferred Reporting Items for Systematic Review and Meta-Analysis
