# Supplementary material for: Prevalence and risk factors of work-related musculoskeletal disorders among Radiographers: a proposed systematic review and meta-analysis protocol
Source: Syst Rev. 2025 Dec 12;15:17. doi: 10.1186/s13643-025-03017-5 (PMC12817418; doi:10.1186/s13643-025-03017-5)
Supplement: Supplementary file 2 — Additional file 2: Example search used for identification of articles on PubMed database. [file 13643_2025_3017_MOESM2_ESM.docx]

Additional file 2:

Example search used for identification of articles on PubMed database

| Search | Search term | MeSH Terms |
| --- | --- | --- |
| Population | | |
| #1 | "Radiographer*"[All Fields] OR "Radiology Technologist"[All Fields] OR "X Ray Technician"[All Fields] OR "X Ray Technologist"[All Fields] OR "radiologi* technologist"[All Fields] OR "imaging professionals"[All Fields] OR "medical imaging technologist"[All Fields] OR "Mammograph*"[All Fields] OR "Mammograph*"[Title] | "diagnostic imaging"[MeSH Subheading] OR "Mammography"[Mesh] |
| Exposure | | |
| #2 | "Workload"[All Fields] OR "Lifting"[All Fields] OR "Moving and Lifting Patients"[All Fields] OR "Ergonomics"[All Fields] OR "awkward posture"[All Fields] OR "human factors"[All Fields] OR “patient transfer” [All Fields] OR “patient handling” [All Fields] | "Workload"[MeSH Terms] OR "Lifting"[MeSH Terms] OR "Moving and Lifting Patients"[MeSH Terms] OR "Ergonomics"[MeSH Terms] OR "patient transfer" [MeSH Terms] |
| Outcome |  |  |
| #3 | "Musculoskeletal Pain" [All fields] OR "work?related musculoskeletal disorder"[All Fields] OR "WRMSD"[All Fields] OR "Musculoskeletal symptoms"[All Fields] OR "cumulative trauma disorders"[All Fields] OR "Repetitive strain injuries"[All Fields] OR "Repetitive motion injuries"[All Fields] OR "Low Back Pain"[All Fields] OR OR "Back Pain"[ All Fields] OR "Low Back Pain"[ All Fields] OR "neck pain"[ All Fields] OR "Shoulder Pain"[ All Fields] OR "Occupational Injuries"[ All Fields] OR "injur*"[All Fields] | "Musculoskeletal Pain"[Mesh] OR "cumulative trauma disorders" [MeSH Terms] OR "Back Pain"[MeSH Terms] OR "Low Back Pain"[MeSH Terms] OR "neck pain"[MeSH Terms] OR "Shoulder Pain"[MeSH Terms] OR "Occupational Injuries"[MeSH Terms] |
| Study Design | | |
| #4 | "epidemiolog*"[All Fields] OR "prevalence*"[All Fields] OR "incidence*"[All Fields] | "epidemiology"[MeSH Terms] OR "incidence"[MeSH Terms] OR "prevalence"[MeSH Terms] |
| Final Search | | |
| #5 | #1 AND #2 AND #3 AND #4 | (("radiographer*"[All Fields] OR "Radiology Technologist"[All Fields] OR "X Ray Technician"[All Fields] OR "X Ray Technologist"[All Fields] OR "radiologi* technologist"[All Fields] OR "imaging professionals"[All Fields] OR "diagnostic imaging"[MeSH Subheading] OR "medical imaging technologist"[All Fields] OR "mammograph*"[All Fields] OR "Mammography"[MeSH Terms] OR "mammograph*"[Title]) AND ((("Workload"[All Fields] OR "Lifting"[All Fields] OR "Moving and Lifting Patients"[All Fields] OR "Ergonomics"[All Fields]) AND "Workload"[MeSH Terms]) OR "awkward posture"[All Fields] OR "Lifting"[MeSH Terms] OR "Moving and Lifting Patients"[MeSH Terms] OR "human factors"[All Fields] OR "Ergonomics"[MeSH Terms] OR "patient transfer"[MeSH Terms] OR "patient transfer"[All Fields] OR "patient handling"[All Fields]) AND ("Musculoskeletal Pain"[All Fields] OR "Musculoskeletal Pain"[MeSH Terms] OR "work?related musculoskeletal disorder"[All Fields] OR "WRMSD"[All Fields] OR "Musculoskeletal symptoms"[All Fields] OR "cumulative trauma disorders"[All Fields] OR "cumulative trauma disorders"[MeSH Terms] OR "Repetitive strain injuries"[All Fields] OR "Repetitive motion injuries"[All Fields] OR "Low Back Pain"[All Fields] OR "Back Pain"[MeSH Terms] OR "Low Back Pain"[MeSH Terms] OR "neck pain"[MeSH Terms] OR "Shoulder Pain"[MeSH Terms] OR "Occupational Injuries"[MeSH Terms] OR "Back Pain"[All Fields] OR "Low Back Pain"[All Fields] OR "neck pain"[All Fields] OR "Shoulder Pain"[All Fields] OR "Occupational Injuries"[All Fields] OR "injur*"[All Fields]) AND ("epidemiology"[MeSH Terms] OR "epidemiolog*"[All Fields] OR "prevalence*"[All Fields] OR "prevalence"[MeSH Terms] OR "incidence*"[All Fields] OR "incidence"[MeSH Terms])) AND ((humans[Filter]) AND (english[Filter])) |

Likewise Scopus, the Cochrane Library, Embase, Web of Science and the Cumulative Index to Nursing and Allied Health Literature (CINAHL) databases will be searched using similar search strings tailored to each database.
